# Observations on Vaccine Inoculation

**Published:** 1802-08-01

**Authors:** 

**Affiliations:** Pimlico, Member of the Royal College of Surgeons


### *Observations on Vaccine Inoculation; communicated by Mr.* THOMAS, *of Pimlico, Member of the Royal College of Surgeons.*

THE highly intereſting diſcovery and candid publication of Dr. Jenner, having undergone the ſeverity of criticiſm, ſtood the teſt of experience, and moreover, been ſtamped with the approbation of Parliament; it may be thought unneceſſary for any private individual to ſay more, with a view of rendering the the vaccine practice general. Indeed, for the honour of the profeffion, I am willing to believe, that if it depended on the medical world, there would *now* be found none illiberal enough to oppofe it. But, (as has been the cafe with all great difcoveries) there are many country people who, even now, queftion and doubt the facts fo fully and fatisfactorily proved by Dr. Jenner and his friends.

I have for many years attended minutely to the practice of Small-pox inoculation, which for the laft feven occupied nearly the whole of my time, having at one period three houfes, in the neighbourhood of Daventry, in the county of Northampton, for the reception of patients under that difeafe. It was a beneficial concern to myfelf, and (if fuccefs can be confidered as a criterion of merit) it was advantageous to my patients, for out of near five thoufand patients, I had the good fortune *not to lose one life.*

My practice did not depend on rules laid down by fome modern authors, but was founded on facts and repeated obfervations, the particulars of which I had reduced to writing, with an intention of fubmitting them to the world, in the form of a publication; but Dr. Jenner's difcovery and account of the vaccine pock, has totally done away the neceffity of fuch an undertaking.

I have feveral times met with men from the counties of Northampton, Buckingham, Oxford, Warwick, and Leicefter, to whom I could not communicate Small-pox infection, and had obferved, that fuch men had been brought up in the farming, dairying, or grazing line of bufinefs. I had alfo obferved, that the inoculated arms of all thofe, whom I termed my unfuccefsful patients, invariably bore the fame appearances, even on repeated trials, (i. e.) an early inflammation, with a hard circumfcribed tumour, which always began to fubfide on the fifth or fixth day, and continued to decreafe till the eighth or ninth, when in general the inflammation entirely difappeared, and the tumour became difcernible only by the touch.

Thefe cafes occurred to me fo often, that I ventured to affure fuch patients, they never would have the Small-pox, though I could not fatisfactorily account for fuch appearances. In my own mind I had imagined they muft have gone through the difeafe before, or that thofe appearances arofe from fome peculiar Idiofyncrafy.

As foon as I had read Dr. Jenner's book, I was convinced thofe patients had had the "Vaccine Pock," and that it would prevent the Small-pox.

Accordingly, I examined my lift of cafes; and thofe I was unfuccefsful with, who refided within twelve or fifteen miles of

Daventry,

Daventry, I called on, with a view to inveftigate minutely every circumftance, and thereby place the matter beyond the poffibility of a doubt. The refult was, that eight people within that diftance fully corroborated the opinion I had formed.

The limits of a paper intended for infertion in a periodical publication, will not allow me to ftate each cafe at length; I fhall therefore only requeft you will have the goodnefs to fuffer me to mention one of them, which, in my opinion, proves that the patient inoculated himfelf with the "Vaccine Pock" *more than thirty years ago*, viz.

A perfon, whofe name is Norris, lives in the parifh of Patifhall, near Forfter's Booth, in the county of Northampton, and follows the bufinefs of a horfe-dealer, applied to me in the fpring of the year 1797, in company with another perfon, whofe name is Haynes, and who lives in the fame parifh. I inoculated them both, and eight more, on the fame day, and with the fame matter; on the third day after, I faw them all again, and the appearance on their arms was fuch as induced me to fay they had all taken the infection; but Norris's arm was more inflamed than any, and I made more fure of his fafety than either of the others. I defired them all to go to my Inoculating Houfe on the eighth day, and told them I would fee them there on that day. When I arrived at the houfe, I found Norris walking in an adjoining field, waiting for me, being afraid to go into the houfe, as he faid his arms were got well; when I looked at them I found there had been a confiderable degree of inflammation, which was then nearly all gone; I alfo difcovered that kind of tumour hardly difcernible but to the touch. Under thefe circumftances I did not advife him to go to the houfe, but inoculated him again in both arms with *matter just taken from a patient who had the natural confluent Small-pox*, and fent him home. On the third day I faw him again, when both his arms put on the fame appearance they had done before; on the fifth day they appeared as thofe do generally on the ninth or tenth day; on the fixth the inflammation began to fubfide, and continued to decreafe till the ninth day, when both arms were well again, having that fmall tumour before defcribed on each of them.

To fatisfy this man, I inoculated him again with more frefh matter, with exactly the fame effect as before; I wifhed him to go to the houfe, and try the effect of a natural infection, but could not prevail on him.

I called on him about two years after, and afked him if ever he remembered milking a cow with a puftule on her teat? (which I explained to him fo that he perfectly underftood me;) and whether he had ever had any fore finger or hand in confe-

 quence?

quence? Before I could finifh my fecond queftion, he pulled off his hat and fhewed me a cicatrix or fcar on his forehead, exactly like thofe we fee on the arms of perfons who had been inoculated with the Small-pox.

He alfo told me, "he had been accuftomed to milk cows for many years; that he remembered feeing fuch a complaint among cows; that having long hair, which ufed to fall over his face while in the act of milking, he was in the conftant habit of throwing it back with his hand; and at one time he was milking a cow with a fore teat, and as ufual throwing back his long hair, he imagined he muft have wiped his finger on a fmall fcratch he had on his forehead, which produced a great fore; he was in confequence obliged to keep his bed, for feveral days was light headed, and the medical man who attended him, declared he never faw fuch a complaint before, nor could he fay what it was." He alfo obferved, "that while he continued fenfible, the inflammation and pain of his head were very great."

I could not poffibly doubt now that the Vaccine pock would prevent the infection of Small-pox; but as moft men are unwilling to countenance a difcovery that would injure them materially, I flattered myfelf that as I had been fo fuccefsful in the treatment of Small-pox Inoculation, no real advantage could arife from Dr. Jenner's difcovery.

I had therefore determined on publifhing "An Account of a New and Succefsful Method of treating the Inoculated Small-pox," the which I had permiffion to dedicate to that ingenious and truly fcientific Surgeon, Mr. John Pearfon; and had alfo determined on an Appendix, where I intended attacking the Vaccine Pock on the maxim of "cui bono," being in my own mind fully perfuaded, both from the account of my own patients, and the obfervation I had made on Dr. Jenner's publication, that the inoculated Small-pox was a lefs painful and milder difeafe than the Vaccine.

With this impreffion on my mind, I prevailed on fome people to be inoculated with the vaccine fluid, propofing at the fame time to inoculate them again with the Small-pox, after they had paffed through the Vaccine Difeafe, not doubting that I fhould thereby have ample proofs to eftablifh the opinion I had formed.

I inoculated fixty* patients, firft with the Vaccine and then with the Small-pox, many of whom were alfo expofed to natural

* I have inoculated feveral hundreds with Vaccine, but only fixty with Small-pox afterwards.

tural infection, and never was able, in any one inftance, to produce the leaft fymptom of Small-pox.

The arms of all bore fimilar appearances to thofe I have already defcribed; and, to my very great difappointment, I found the Vaccine-pock fo fafe and mild a difeafe (none of my patients fuffered a day's illnefs) that I became a convert; was obliged to drop the idea of publifhing my manufcript, and in a very fhort time compelled to fhut up my Inoculating Houfes, there being no longer an occafion for them.

Thus you will obferve, that I was completely foiled in my attempt to prevent the introduction of Vaccine Inoculation; for the very ftep I took with a view to fupprefs it, only ferved to bring it into general eftimation in that part of the country; and, I believe, if people could diveft themfelves of prejudice, and be content with a fair and impartial inveftigation of facts, that loathfome and terrible difeafe the Small-pox, would foon be unknown among mankind.

As an individual, no one has greater reafon to lament the introduction of Vaccine, and the confequent abolition of Small-pox Inoculation than myfelf; it has not only been the means of depriving me of a very comfortable fupport for my family, but abfolutely compelled me to leave a place and connection I much valued, confequently it muft be allowed that I can have no other motive for obtruding my obfervations on the public but the *public good*.

*July* 5, 1802.

